# The Effects of Fineness and TEA-Based Chemical Admixture on Early Strength Development of Concrete in Construction Site Applications

**DOI:** 10.3390/ma13092027

**Published:** 2020-04-26

**Authors:** Taegyu Lee, Jaehyun Lee, Hyeonggil Choi, Dong-Eun Lee

**Affiliations:** 1 Technology Research & Development Institute, Daelim Industrial, Jongno-Gu, Seoul 03152, Korea; 2 School of Architecture and Civil Engineering, Kyungpook National University, 80 Daehakro, Bukgu, Daegu 41566, Korea

**Keywords:** cement fineness, fine ordinary Portland cement, TEA-based chemical admixture, concrete strength, early strength development

## Abstract

This study examines effects of cement fineness and chemical admixtures of early strength agents on the early strength development of concrete. Three cement types were selected, namely ASTM type-I ordinary Portland cement (OPC), fineness ordinary Portland cement (FOPC), and ASTM type-III early Portland cement (EPC), and the mixing proportions of concrete were set by adding a triethanolamine-based chemical admixture to FOPC. The evaluation items considered in this study included raw material analysis, compressive strength, and maturity (D∙h). The time required for the development of concrete strength of 5 MPa in the three cement types was estimated and compared. The results revealed that using FOPC enhances the strength development of concrete owing to its higher fineness and SO_3_ content compared to OPC. In addition, it has been observed that using both FOPC and TCA yields a similar performance to that observed using EPC, in light of the improved early strength development at low temperatures.

## 1. Introduction

Concrete forms a structural matrix by combining cement, water, fine aggregate, coarse aggregate, and admixture. The strength of concrete is developed by the homogeneous bonding force of each material, and the primary influential factor of the strength development is cement. Cement is condensed and cured as it stabilizes while releasing heat energy through its hydration reaction with water [1,2,3]. Concrete features excellent compressive strength development, while facilitating easy attachment of reinforcement bars. In addition, the strong alkali performance of concrete ensures durability by limiting the corrosion of reinforcing bars. In addition, concrete is easily available for purchase and use. Additionally, the use of concrete is favored in construction sites owing to its excellent workability prior to condensation and hardening. Concrete hardens and develops strength when its fluidity decreases. This is an important target of quality control, as a critical path for construction sites [4].

Moreover, the most economical and efficient process control is pursued in construction sites, which is closely related to the strength development period of concrete. Concrete remains in formwork until its strength development achieves sufficient structural stability, and each country has different criteria for compressive strength. These include the minimum time [5,6,7] and minimum strength [8,9] required for formwork removal. Concrete strength of 5 MPa or higher is considered a minimum strength criterion to ensure structural stability.

As concrete is significantly affected by temperature during the hydration process of cement, its strength development tends to decrease at low temperatures. There are cases when strength development becomes impossible if the hydration reaction stops for a certain period. Considering these characteristics, many studies have reported on the early strength development of concrete at various curing temperatures [10,11,12].

Ordinary Portland cement (OPC) has been primarily used as a binder because it exhibits stable strength development at temperatures of 15 °C or higher. Its strength development, however, tends to be delayed at low temperatures. Consequently, cases in which strength development was examined using early strength cement and early strength chemical admixture to improve the strength development of cement through hydration have been continuously reported [13,14,15,16,17,18].

Frigione et al. [19,20,21] reported that early strength development can be achieved through the rapid reaction between cement and water by increasing the fineness of the cement.

It has been observed that the hydration degree of cement increases with a reduction in its particle size. Klein and Metha [22] presented the possibility of early strength development for the CaO-Al_2_O_3_-SO_3_ system due to the generation of various hydrates according to the Al_2_O_3_/SO_3_ and CaO/SO_3_ molar ratios. Lee et al. [23,24] examined the effect of fineness and SO_3_ content on the strength development of fine ordinary Portland cement (FOPC) at early ages.

It has been observed that commercialized fine ordinary Portland cement (FOPC), which improved the OPC powder and SO_3_ contents, demonstrates enhanced early strength development in the 13–15 °C temperature range. ASTM C150 [25] specifies 3.1% as the optimum SO_3_ concentration in cement to ensure sufficient early strength of OPC.

Hewlett et al. [26,27,28] proposed the use of a high-performance superplasticizer added to an early accelerating agent to improve early strength of OPC. Additionally, they presented results pertaining to early strength development of triethanolamine (TEA)-based chemical admixtures, which facilitates acceleration of the hydration of tricalcium aluminate (C3A), thereby contributing to OPC hydration.

However, these extant studies mostly dealt with mortar-based materials and lacked consideration of quality control aspects, such as those pertaining to the economic efficiency of mixing, batch plant productivity, and in-field workability during application in construction sites. In addition, it is necessary to examine the early strength of various commercialized cements in order to ensure consistent quality in mass production and optimized mix design in construction sites.

In this study, various binders, such as OPC, early Portland cement (EPC), and FOPC, were selected based on the C24 (characteristic value of concrete 24 MPa) concrete mix, which is produced in ready-mixed concrete factories. Their influence on early strength development at different curing temperatures was examined. In addition, polycarboxylate (PC)-based superplasticizers with early strength agents were added and their influence on strength development was analyzed.

## 2. Materials and Methods

### 2.1. Materials

Table 1 shows the chemical compositions of the used binders by X-ray fluorescence (XRF, Axios PW 4400, Malvern Panalytical, Seongnam-si, Korea) analysis. The concrete binders ASTM type-I OPC with density equal to 3150 kg/m^3^ and fineness of 330 m^2^/kg, FOPC with density equal to 3130 kg/m^3^ and fineness of 380 m^2^/kg, and ASTM type-III EPC with density equal to 3160 kg/m^3^ and fineness of 488 m^2^/kg were considered in this study.

**Figure 1:**
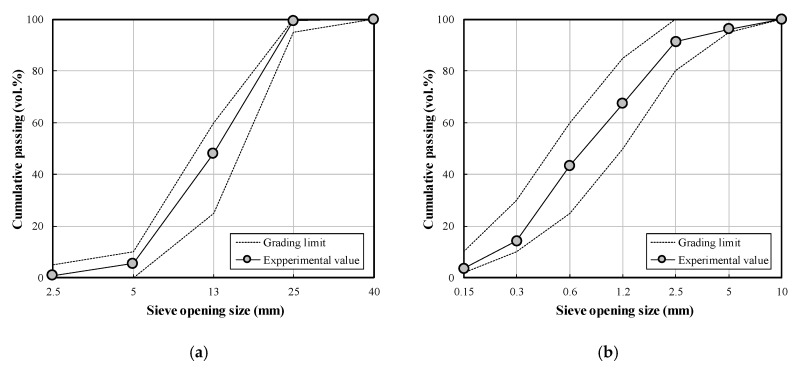
Gradation sieve analysis curves of aggregates used: (**a**) coarse aggregates; (**b**) fine aggregates.

Figure 1 shows the gradation sieve analysis curves of the fine and coarse aggregates used. As for fine aggregate, washed sea sand (fineness: 2.01, density: 2600 kg/m^3^, absorption: 0.79%) and crushed sand (fineness: 3.29, density: 2570 kg/m^3^, absorption: 0.87%) were mixed and used. The fineness of the mixed fine aggregate was 2.84. For the coarse aggregate, crushed granite aggregate (size: 25 mm, density: 2600 kg/m^3^, absorption: 0.76%) was used.

As for chemical admixtures, a PC-based admixture (polycarboxylic acid group) and a PC-based admixture with added sodium nitrate (NaNO_3_) and sodium sulfate (Na_2_SO_4_) based on triethanolamine (TEA) (hereafter, TEA-based chemical admixture) were used.

### 2.2. Experimental Plan and Mix Proportions

Table 2 lists the experimental plan followed in this study. As for the concrete strength, C24, which is most commonly used in construction sites, was selected. The water/cement (W/C) ratio was fixed at 0.50, the unit water content was 165 kg/m^3^, and the total amount of cement was 330 kg/m^3^. As for the types of cement, OPC, FOPC, and EPC were selected. For chemical admixtures, PC-based superplasticizer was used. Additionally, a TEA-based chemical admixture was used for FOPC because it was expected to increase the chemical effect of FOPC (refer to Lee et al. [23,29]).

**Figure 2:**
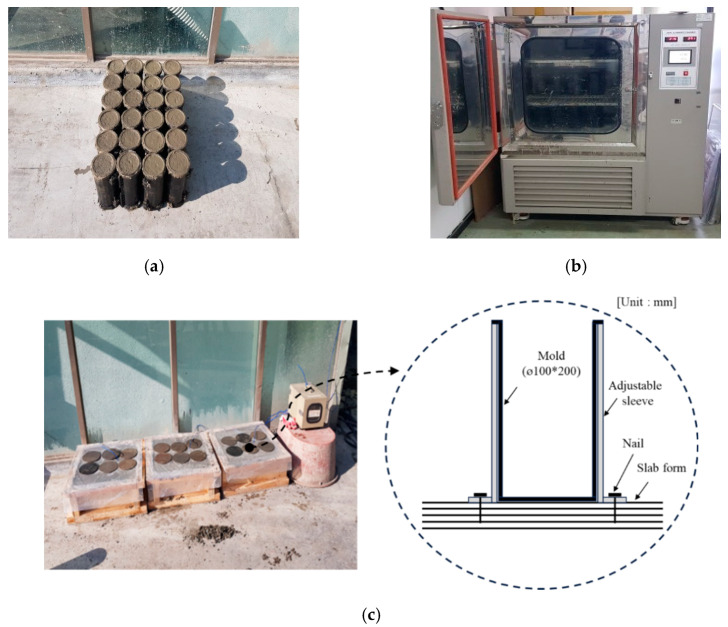
Photographs of concrete curing methods: (**a**) air curing; (**b**) chamber curing; (**c**) cast-in-place (slab-embedded specimens for outdoor curing).

To establish concrete curing temperatures, the outdoor (average temperature = 12 °C), chamber (13 °C), and cast-in-place (slab-embedded specimens for outdoor curing) conditions were set as illustrated in Figure 2. The compressive strength and maturity (D∙h) were measured as evaluation items. Finally, the 5 MPa development time was estimated.

Table 3 shows the mixing proportions of concrete. The unit water content of concrete was set to 165 kg/m^3^, considering the water-reducing ratio of the PC-based admixture. Fine aggregate, washed sea sand, and crushed sand were mixed at a 4:6 ratio. As for the properties of concrete, the slump was set to 180 ± 25 mm and the air content to 4.5 ± 1.5% to secure sufficient workability for field construction.

### 2.3. Test Methods

#### 2.3.1. Properties of Raw Materials

Table 4 shows the test plan for the properties of the raw materials. In this study, an attempt was made to examine the effects of various types of cement on early strength development. The physical and chemical properties of cement may affect the properties and strength development of concrete. For the raw material analysis, particle size distribution was evaluated in accordance with ASTM C204 [30], scanning electron microscope analysis in accordance with ASTM C1723 [31], XRD in accordance with ASTM C1365 [32], and heat of hydration of cement in accordance with ASTM C1702 [33]. Scanning electron microscopy was performed using the Genesis-2020 microscope (Emcrafts, Gwangju-si, Korea) at 20 kV voltage. X-ray diffraction was performed using X’pert3 Powder PW 3050 (Malvern Panalytical, Seongnam-si, Korea) at 60 kV and 50 mA voltage and current ratings, respectively. Analysis pertaining to heat of hydration of cement was performed using MMC-511SV6 (6 CH, Tokyo Riko, Tokyo, Japan).

#### 2.3.2. Fresh and Hardened Properties of Concrete

Table 5 shows the test methods for fresh and hardened properties of concrete. For fresh concrete, the slump was evaluated in accordance with ASTM C143/C143M [34] and the air content was tested in accordance with ASTM C231/C231M-17a [35]. As for the properties of fresh concrete, the initial properties and properties after 60 min of curing were evaluated considering workability from ready-mixed concrete production to on-site pouring.

The compressive strength of concrete was calculated by measuring the maximum load at the target age using a 300 ton universal test machine after fabricating Ø 100 × 200 mm specimens in accordance with ASTM C873 [36] and ASTM C39/C39M [37]. The compressive strength of concrete was calculated as the average value obtained for three test specimens.

#### 2.3.3. Maturity of Concrete

Figure 2 shows images of the concrete curing test methods. Concrete was cured using air, chamber (13 °C), and cast-in-place methods. The cast-in-place method was conducted in accordance with ASTM C873/C873M [38].

This method examines the compressive strength of concrete by simulating the site slab and reproducing the temperature history of the structure. In this study, 500 × 500 × 210 mm^3^ mock-ups were fabricated, and ø 100 × 200 mm cylinder molds were embedded using sleeves. The molds were fixed to the formwork using nails to minimize deformation due to their movement during concrete pouring.

The hydration heat of concrete was measured by embedding K-type thermocouples in the center of each specimen. The maturity of concrete was calculated using Equation (1), based on the hydration heat measured in accordance with ASTM C1074 [38]:

(1) $$M\left(t\right)=\sum (T_{a}-T_{0})\Delta t$$

where *M*(*t*) = the temperature–time factor at age t, shown as degree days or degree hours; *∆t* = a time interval, shown as days or hours, *T_a_* = average concrete temperature during time interval *∆t* in °C; and *T_o_* = datum temperature in °C.

## 3. Results and Discussion

### 3.1. Analysis of Properties of Cement Raw Materials

**Figure 3:**
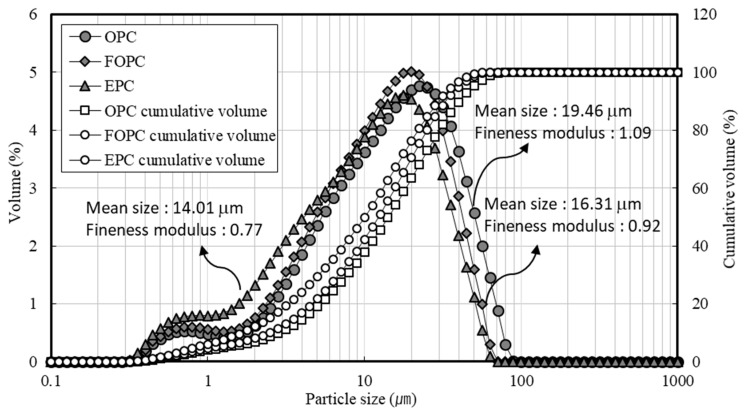
Particle size distribution for OPC, FOPC, and EPC.

**Figure 4:**
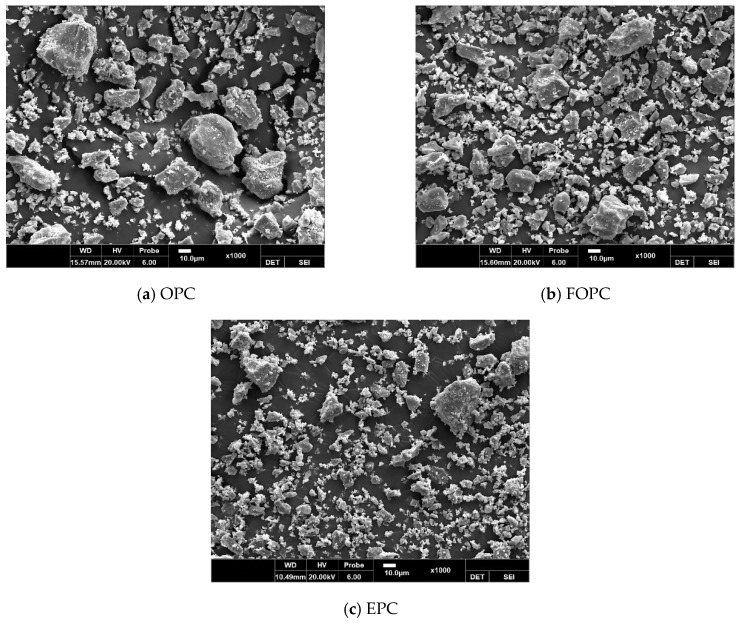
Scanning electron microscope micrographs: (**a**) OPC; (**b**) FOPC; (**c**) EPC.

Figure 3 shows the particle size distribution of OPC, FOPC, and EPC used in this study. OPC has a mean size of 19.46 μm and a fineness modulus of 1.09, while FOPC has a mean size of 16.31 μm and a fineness modulus of 0.92. EPC has a mean size of 14.01 μm and a fineness modulus of 0.76. EPC has the highest fineness, followed by FOPC and OPC. As for the detailed grain shape of the cement, OPC had many large particles and EPC had many fine particles, as shown in Figure 4. OPC and FOPC similarly exhibited the shape of an elliptical polyhedron for particles larger than 10 μm, and EPC showed fine and irregular particles. In the case of FOPC, fine and irregular particles were distributed among the elliptical polyhedrons.

**Figure 5:**
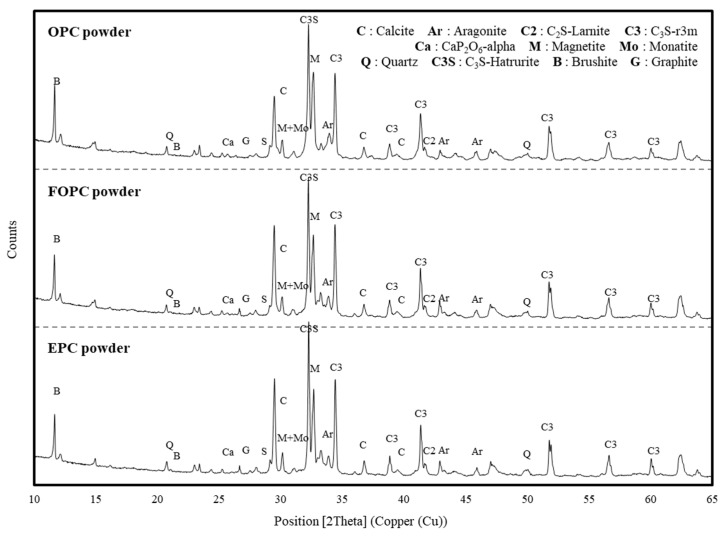
X-ray diffraction patterns obtained for OPC, FOPC, and EPC.

Figure 5 depicts X-ray diffraction patterns pertaining to OPC, FOPC, and EPC considered in this study. It was confirmed that all cement types mainly comprise C_3_S (3CaO∙SiO_2_), C_2_S (2CaO∙SiO_2_), C_3_A (3CaO∙Al_2_O_3_), and C_4_AF (4CaO∙Al_2_O_3_∙Fe_2_O_3_) minerals. EPC contains more C_3_S crystal peaks compared to both OPC and FOPC. The amount of C_3_S calculated by Bogue’s equation [39] on X-ray fluorescence analysis of OPC, FOPC, and EPC in Table 1 equaled 50.68%, 48.72%, and 56.47%, respectively.

### 3.2. Fresh and Hardened Properties of Concrete

Table 6 shows the fresh properties of concrete. All the concrete mixes met the target slump of 180 ± 25 mm. In the case of the FOPC mix, the slump decreased by 35 mm, showing a significant reduction. The air content met the target of 4.5 ± 1.5% for all the concrete mixes and a slight reduction occurred after 60 min.

**Figure 6:**
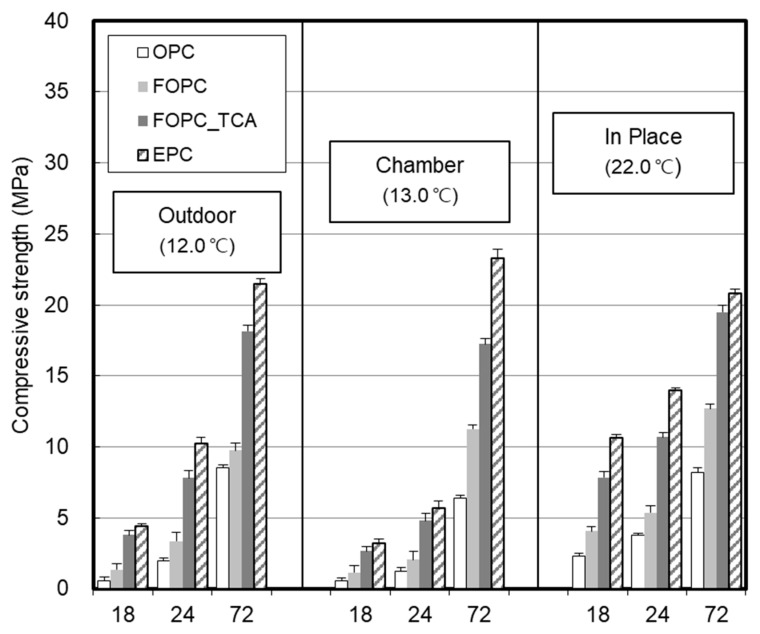
Early compressive strength development in concrete with time.

Figure 6 shows early compressive strength development in concrete with time. In the case of the OPC-based concrete, it took more than 24 h to develop 5 MPa at temperatures higher than 22 °C and approximately 72 h at 13 °C.

The FOPC-based concrete exhibited slightly higher strength compared to OPC; the strength development effect was larger at temperatures higher than 22 °C than at lower temperatures. In the case of the concrete that used FOPC and TCA, the 5 MPa strength development occurred within 24 h at 13 °C. The strength development of the concrete that used FOPC and TCA further increased as the curing temperature increased, making it possible to develop 5 MPa within an 18 h.

FOPC_TCA and EPC exhibited similar early strength performance within 24 h at low temperatures, but EPC showed higher strength after 72 h. At temperatures higher than 22 °C, FOPC_TCA and EPC could develop 5 MPa within 18 h. They exhibited similar strength after 72 h.

### 3.3. Temperature History and Maturity of Concrete

**Figure 7:**
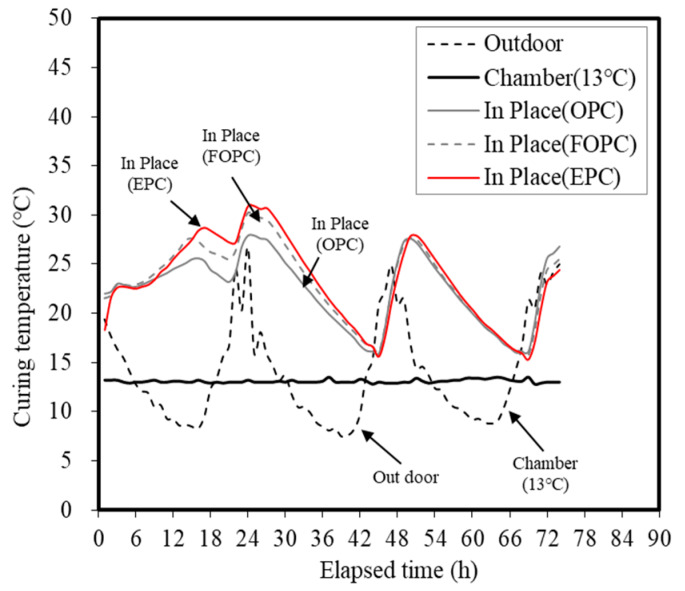
Temperature history of concrete for different curing methods.

**Figure 8:**
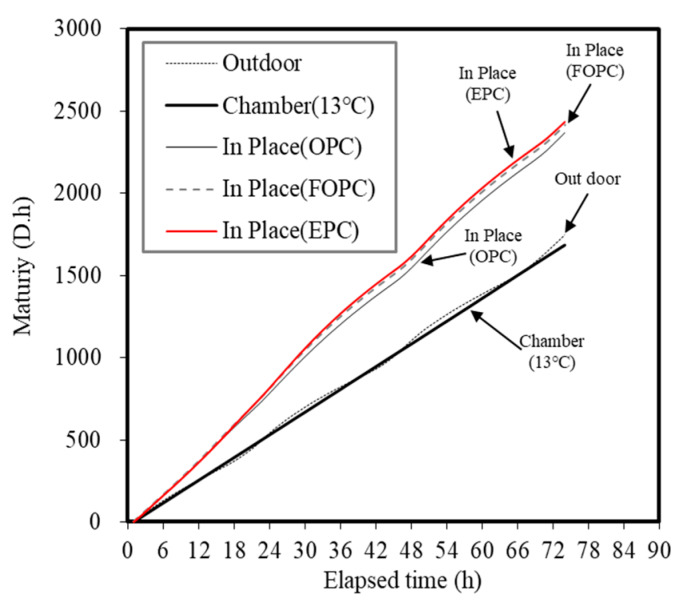
Concrete maturity trends for concrete when using different curing methods.

Figure 7 and Figure 8 show the temperature history and maturity of each curing method at elapsed time points. The temperature history of concrete was maintained at 13 °C when a constant temperature and humidity chamber was used. In the case of the outdoor conditions, diurnal temperature changes occurred and repeated temperature patterns between 8 and 28 °C were observed. As for the specimens embedded in the concrete mock-ups, the temperature increased until 30 h due to the hydration heat of concrete. Afterwards, repeated temperature patterns occurred in line with the outdoor temperature history.

Results obtained in this study reveal that when using a small test specimen measuring ø 100 × 200 mm, temperature histories pertaining to each mixture are nearly similar in the ± 2 °C range of the outdoor and chamber curing temperatures. Therefore, this was suggested as the curing temperature for the outdoor and chamber samples (13 °C). When using the cast-in-place curing method, it was confirmed that the temperature history differs from the curing temperature corresponding to the outdoor method using a mock-up member measuring ø 500 × 500 × H210 mm^3^. Accordingly, five temperature histories and accumulated temperatures corresponding to the outdoor, chamber (13 °C), and cast-in-place (OPC, FOPC, EPC) curing methods were determined.

The maturity of concrete linearly increased over time at all curing levels. Similar maturity increase patterns were observed under both outdoor and chamber (13 °C) conditions. The cast-in-place curing condition exhibited significantly higher values compared to the outdoor and chamber (13 °C) conditions, suggesting that the influence of the temperature history of a structure can favor strength development. Among cement types considered in this study, EPC demonstrated the highest maturity, followed by FOPC and OPC. It has been confirmed that cases affected by the temperature history of a structure exhibit higher strength development compared to small-specimen conditions. As observed, EPC- and FOPC-based concrete mixtures demonstrated greater potential for early strength development.

**Figure 9:**
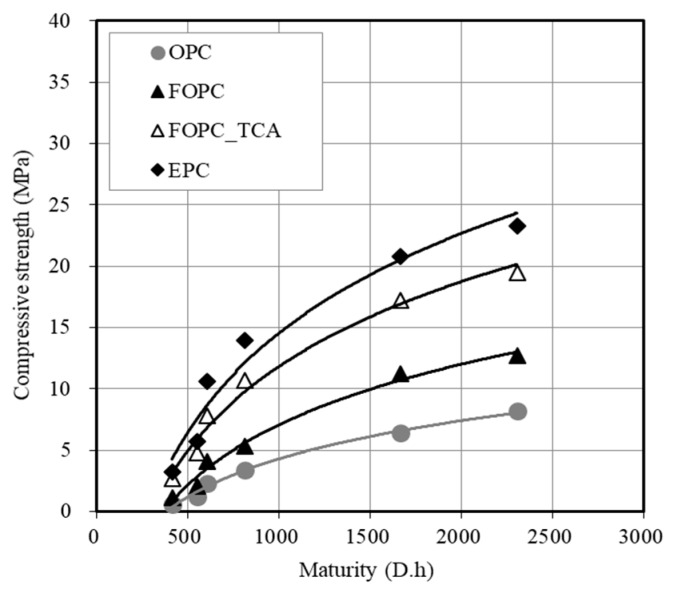
Comparison of maturity and early strength of concrete.

Figure 9 shows the comparison of the maturity and early strength of concrete. The maturity to develop a concrete compressive strength of 5 MPa was found to be 1364 D∙h for OPC, 748 D∙h for FOPC, 504 D∙h for FOPC_TCA, and 443 D∙h for EPC. The reduction rate of the maturity for 5 MPa strength development compared to OPC was 45% when FOPC was used. FOPC_TCA (63%) and EPC (68%) exhibited similar reductions.

### 3.4. Analysis of Factors for Early Strength Development of Concrete

**Figure 10:**
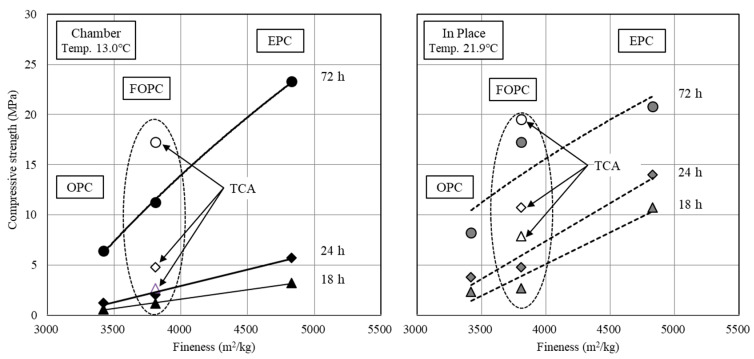
Comparison of cement fineness and early strength of concrete.

Figure 10 shows the comparison of cement fineness and the early strength of concrete. There was a linear relationship between the cement fineness and the compressive strength under the low-temperature curing condition of 13 °C. The compressive strength increased as the cement fineness increased, and the slope significantly increased after 72 h. In the case of FOPC_TCA, the compressive strength development rate was more than twice that of FOPC and its effect was larger within 24 h. Although FOPC_TCA was promising for securing the early compressive strength of 5 MPa, its effect was found to be slightly lower compared to EPC in terms of long-term strength.

At a temperature higher than 20 °C, a linear relationship with the cement fineness was observed similarly to the under 13 °C curing condition, but the increment was larger. In addition, the compressive strength development rate significantly increased after 72 h, resulting in smaller differences from EPC. This tendency was more noticeable when TCA was mixed. These results confirmed that the effect of increasing the fineness increased as the temperature increased, and that the effect of adding TCA was excellent at low temperatures [40,41,42].

**Figure 11:**
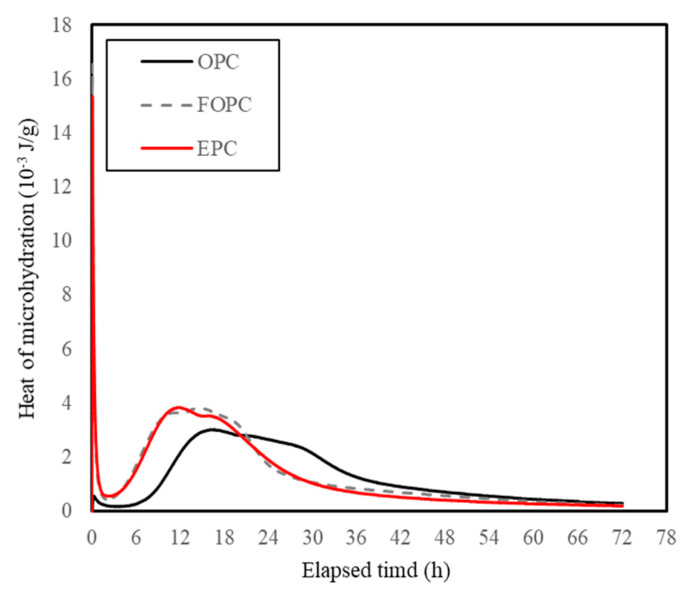
Heat of micro-hydration observed for OPC, FOPC, and EPC.

**Figure 12:**
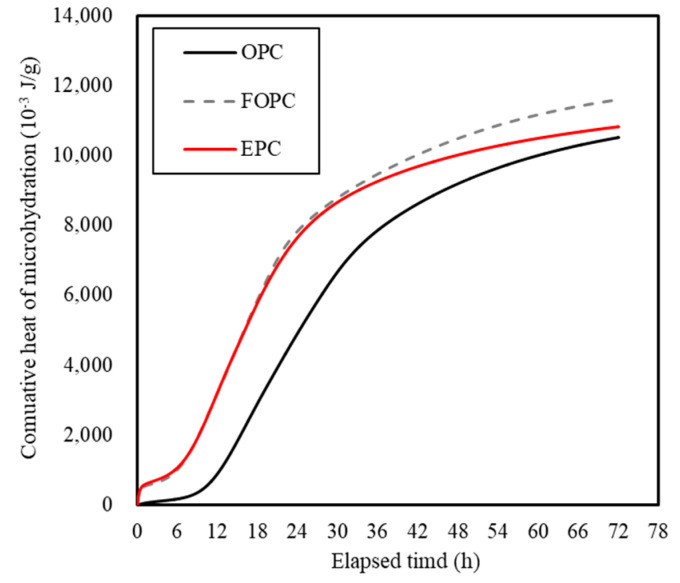
Cumulative heat of micro-hydration observed for OPC, FOPC, and EPC.

This difference in fineness is a major factor influencing the difference in calories due to cement hydration. Figure 11 depicts trends concerning heat of micro-hydration observed for OPC, FOPC, and EPC. As observed, FOPC and EPC demonstrate higher values compared to OPC during the early age of up to 24 h. Figure 12 depicts trends concerning the cumulative heat of micro-hydration observed for OPC, FOPC, and EPC. As observed, the said cumulative values for FOPC and EPC are more than twice the value corresponding to OPC.

The SO_3_ content for FOPC and EPC ranged from 3% to 4%, which is 107–129% higher compared to OPC. These results are similar to Mohammed’s report [43], which found that the compressive strength significantly increased after 48 h when the SO_3_ content ranged from 3.0% to 3.2%. In addition, Lee reported that the early strength increased when the cement fineness was increased, while the SO_3_ content was increased to 3.1 [23,24,25]. In the scope of this study, we found that the effect of additionally using TCA on the early strength of concrete increases with the SO_3_ content of 4%.

**Figure 13:**
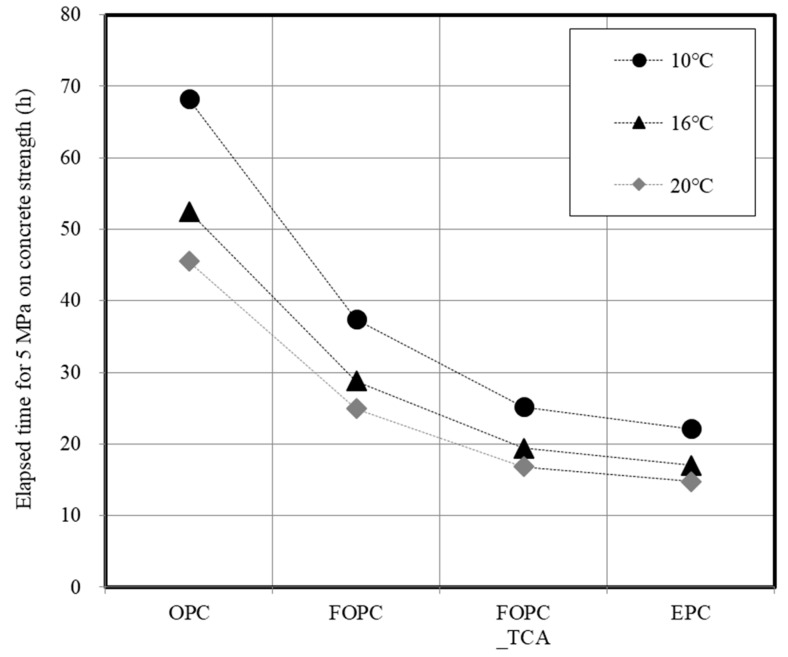
Elapsed time for development of 5 MPa strength in concrete using maturity method for different curing temperatures.

Figure 13 depicts trends concerning variation in the time required for development of 5 MPa strength in concrete using the maturity method in accordance with the curing temperature.

In construction sites, the critical path of construction process management is the period of vertical formwork. The removal time of vertical formwork is based on ACI (10 °C, 12 h), BS EN (16 °C, 12 h), and Asian (Korea, Japan, 5-MPa strength development) standards [5,6,7,8,9]. This study refers to the Asian standard, which proposes use of a detailed value to evaluate the effect of concrete on early strength development. Through analysis of the relationship between maturity and compressive strength development, the time required for development of 5 MPa strength was calculated in accordance with the average curing temperature.

EPC, which had the highest cement fineness and SO_3_ content, exhibited the shortest elapsed time for 5 MPa development, followed by FOPC and OPC. As the temperature decreased, the time reduction effect became larger. In the case of FOPC_TCA, the elapsed time for 5 MPa significantly decreased to a level equivalent to that of EPC.

## 4. Conclusions

In this study, the effects of the fineness and TEA-based chemical admixture on the early strength development of concrete were examined. The results are summarized as follows.

1) When evaluating the compressive strength of concrete, it was observed that the use of FOPC increases the early strength development effect at high temperatures compared to lower ones. At a curing temperature of 13 °C, the development of compressive strength of FOPC_TCA was observed to increase significantly compared to FOPC. FOPC_TCA demonstrated lower overall compressive strength compared to EPC, but the overall difference observed over a 24 h period was negligible. With increase in curing temperature, the compressive strength development of FOPC_TCA was observed to increase significantly and tended to decrease the difference as compared to EPC.
2) The evaluation of the maturity of concrete revealed that OPC exhibited the highest maturity to develop a concrete compressive strength of 5 MPa, followed by FOPC, FOPC_TCA, and EPC, respectively. The reduction rate of the maturity for 5 MPa concrete strength development compared to OPC was 45% when FOPC was used. FOPC_TCA (63%) and EPC (68%) exhibited similar values.
3) There was a linear relationship between the cement fineness and the compressive strength regardless of temperature conditions, and it was found that higher temperatures had a larger influence on strength development. FOPC, with a higher fineness than OPC, showed a larger impact on strength development at high temperatures, but it is considered that the use of TCA will lead to a strength development rate equivalent to that of EPC, even at low temperatures.
4) For FOPC and EPC, the SO_3_ content ranged from 3% to 4%, which was 107% to 129% higher than that of OPC. As for the SO_3_/Al_2_O_3_ ratio, FOPC exhibited a 116.1% higher value and EPC a 132.2% higher value than that of OPC, indicating that they were effective in developing early strength.

The FOPC that was used in the scope of this study can contribute to improving the strength development of concrete, because its fineness and SO_3_ content are higher than those of OPC. In addition, the use of both FOPC and TCA can achieve performance similar to that of EPC. because it can improve early strength development, even at low temperatures. Considering construction site productivity and economic efficiency, the use of FOPC is considered to have a marked effect compared to OPC, but its economic efficiency compared to EPC needs to be examined in more detail. The results of this study are expected to be used as the basic data for the performance design of concrete mixes to shorten construction periods on construction sites.

## Figures and Tables

**Table 1:** Chemical composition of binders used in this study, as observed via X-ray fluorescence (XRF) analysis.

Materials	Chemical Compositions (%)								L.O.I. ^4^
	CaO	SiO_2_	Al_2_O_3_	MgO	Fe_2_O_3_	SO_3_	K_2_O	Others	
OPC ^1^	61.44	20.33	4.72	2.95	3.42	2.9	0.95	1.62	1.67
FOPC ^2^	60.34	19.82	4.85	3.83	3.30	3.13	1.08	0.63	3.02
EPC ^3^	61.00	19.22	4.51	4.14	3.35	3.73	1.04	0.19	2.82

^1^ OPC: ordinary Portland cement; ^2^ FOPC: fine ordinary Portland cement; ^3^ EPC: early Portland cement; ^4^ L.O.I.: loss on ignition.

**Table 2:** Experimental plan.

W/C ^1^	Cement Type	Unit Weight of Cement (kg/m^3^)	Chemical Admixture	Curing Temperature (°C)	Evaluation Item
0.50	OPC FOPC FOPC EPC	330 330 330 330	PC ^2^ PC TCA ^3^ PC	Outdoor ^4^ Chamber (13 °C) Cast in Place ^5^	Slump (mm) Air contents (%) Compressive strength - Cylinder Mold(Ø100 × 200) - 18 h, 24 h, 72 h Maturity (D∙h)

^1^ W/C: water/cement ratio; ^2^ PC: polycarboxylate; ^3^ TCA (TEA-based chemical admixture); ^4^ Outdoor: average temperature = 12 °C; ^5^ Cast in place (embedded specimens into the slab at curing the outdoor).

**Table 3:** Mixing proportions of concrete.

Series	W/C ^1^	S/a ^2^ (%)	Unit Weight (kg/m^3^)				PC ^7^ (B×%)	TCA ^8^ (B×%)
			W ^3^	C ^4^	S ^5^	G ^6^		
OPC	0.50	50.0	165	330	908	919	3.30	
FOPC	0.50	50.0	165	330	908	919	3.30	
FOPC_TCA	0.50	50.0	165	330	908	919	-	3.30
EPC	0.50	50.0	165	330	908	919	3.30	

^1^ W/C: water/cement; ^2^ S/a: sand/aggregates; ^3^ W: water; ^4^ cement; ^5^ crushed sand; ^6^ G: gravel; ^7^ PC: polycarboxylic superplasticizer-based type admixture; ^8^ TCA: TEA-based chemical admixture (polycarboxylic superplasticizer-based type with accelerator agent, TEA).

**Table 4:** Test plan for raw material properties.

Items	Materials	Evaluation Items	Test Methods
Raw material analysis	OPC FOPC EPC	Particle size distribution (%)	ASTM C204
		Scanning electron microscope	ASTM C1723
		X-ray diffraction	ASTM C1365
		Heat of hydration	ASTM C1702

**Table 5:** Test methods for fresh and hardened properties of concrete.

Type	Evaluation Item	Test Method
Concrete	Slump (mm)	ASTM C143
	Air contents (%)	ASTM C231
	Compressive strength (MPa)	ASTM C873
		ASTM C39

**Table 6:** Fresh properties of concrete.

Mix No.	Slump (mm)		Air contents (%)	
	Initial	After 60 m	Initial	After 60 m
OPC	200	185	5.4	5.0
FOPC	200	165	4.4	4.0
FOPC_TCA	205	170	5.8	5.4
EPC	195	175	5.5	5.0

## References

[1] Mehta P., Monteiro P. (2006). Concrete: Microstructure, Properties, and Materials.

[2] Juilland P., Gallucci E., Flatt R., Scrivener K. (2010). Dissolution theory applied to the induction period in alite hydration. Cem. Concr. Res..

[3] Barnes P., Bensted J. (2002). Structure and Performance of Cements.

[4] Gartner E.M., Young J.F., Damidot D.A., Jawed I., Bensted J., Barnes P. (2002). Composition of cement phases. Structure and Performance of Cements.

[5] ACI 347-04 (2005). Guide to Formwork for Concrete.

[6] Thomas Telford Services Ltd (1993). Ceb-Fip Model Code 1990: Design Code.

[7] BS EN 13670:2009 (2010). Execution of Concrete Structures.

[8] Architectural Institute of Korea (2009). Korea Architectural Standard Specification Reinforced Concrete Work, KASS 5.

[9] Architectural Institute of Japan (2009). Japanese Architectural Standard Specification Reinforced Concrete Work JASS 5.

[10] Lidstrom L., Westerberg B. (2003). Fine ground cement in concrete-properties and prospects. ACI Mater. J..

[11] Kadri E.H., Duval R. (2002). Effect of ultrafine particles on heat of hydration of cement mortars. ACI Mater. J..

[12] Korpa A., Kowald T., Trettin R. (2008). Hydration behaviour, structure and morphology of hydration phases in advanced cement-based systems containing micro and nanoscale pozzolanic additives. Cem Concr Res..

[13] Popescu C., Muntean M., Sharp J. (2003). Industrial trial production of low energy belite cement. Cem. Concr. Compos..

[14] Winnefeld F., Martin L., Müller C., Lothenbach B. (2017). Using gypsum to control hydration kinetics of CSA cements. Constr. Build. Mater..

[15] Gartner E. (2004). Industrially interesting approaches to low CO_2_ cements. Cem. Concr. Res..

[16] Zajac M., Skocek J., Bullerjahn F., Haha M. (2016). Effect of retarders on the early hydration of calcium-sulpho-aluminate (CSA) type cements. Cem. Concr. Res..

[17] Wang P., Li N., Xu L. (2017). Hydration evolution and compressive strength of calcium sulphoaluminate cement constantly cured over the temperature range of 0 to 80 °C. Cem. Concr. Res..

[18] Trauchessec R., Mechling J., Lecomte M., Roux A., Rolland B. (2015). Hydration of ordinary Portland cement and calcium sulfoaluminate cement blends. Cem. Concr. Compos..

[19] Frigione G., Marra S. (1976). Relationship between particle size distribution and compressive strength in Portland cement. Cem. Concr. Res..

[20] Osbaeck B., Johansen V. (1989). Article size distribution and rate of strength development of Portland cement. J. Am. Ceram. Soc..

[21] Bentz D. (2010). Blending different fineness cements to engineer the properties of cement-based materials. Mag. Concr. Res..

[22] Mehta P., Klein A. (1965). Formation of ettringite by hydration of a system containing an anhydrous calcium sulfoaluminate. J. Am. Ceram. Soc..

[23] Lee J., Lee T. (2019). Influences of Chemical Composition and Fineness on the Development of Concrete Strength by Curing Conditions. Materials.

[24] Lee J., Lee T. (2019). Effects of High CaO Fly Ash and Sulfate Activator as a Finer Binder for Cementless Grouting Material. Materials.

[25] ASTM C150 C150M-19a (2019). Standard Specification for Portland Cement. American Society of Testing and Materials.

[26] Hewlett P. (1998). Lea’s Chemistry of Cement and Concrete.

[27] Heren Z., Ölmez H. (1996). The influence of ethanolamines on the hydration and mechanical strength properties of Portland cement. Cem. Concr. Res..

[28] Aggoun S., Cheikh-Zouaoui M., Chikh N., Duval R. (2008). Effect of some admixtures on the setting time and strength evolution of cement pastes at early ages. Constr. Build. Mater..

[29] Lee T., Lee J., Kim Y. (2020). Effects of admixtures and accelerators on the development of concrete strength for horizontal form removal upon curing at 10 °C. Constr. Build. Mater..

[30] ASTM C204 (2018). Standard test methods for fineness of hydraulic cement by air-permeability apparatus. American Society of Testing and Materials.

[31] ASTM C1723-16 (2010). Standard guide for examination of hardened concrete using scanning electron microscopy. American Society of Testing and Materials.

[32] ASTM C1365 (2018). Standard Test Method for Determination of the Proportion of Phases in Portland Cement and Portland-Cement Clinker Using X-Ray Powder Diffraction Analysis. American Society of Testing and Materials.

[33] ASTM C1702 (2015). Standard Test Method for Measurement of Heat of Hydration of Hydraulic Cementitious Materials Using Isothermal Conduction Calorimetry. American Society of Testing and Materials.

[34] ASTM C143/C143M REV A. (2015). Standard Test Method for Slump of Hydraulic-Cement Concrete. American Society of Testing and Materials.

[35] ASTM C231/C231M-17a (2017). Standard Test Method for Air Content of Freshly Mixed Concrete by the Pressure Method. American Society of Testing and Materials.

[36] ASTM C873/C873M (2015). Standard Test Method for Compressive Strength of Concrete Cylinders Cast in Place in Cylindrical Molds. American Society of Testing and Materials.

[37] ASTM C39/C39M (2018). Standard Test Method for Compressive Strength of Cylindrical Concrete Specimens. American Society of Testing and Materials.

[38] ASTM C1074 (2019). Standard Practice for Estimating Concrete Strength by the Maturity Method. American Society of Testing and Materials.

[39] Bogue R. (1929). Calculation of the compounds in portland cement. Indus. Eng. Chem..

[40] Cheung J., Jeknavorian A., Roberts L., Silva D. (2011). Impact of admixtures on the hydration kinetics of Portland cement. Cem. Concr. Res..

[41] Katsioti M., Tsakiridis P., Giannatos P., Tsibouki Z., Marinos J. (2009). Characterization of various cement grinding aids and their impact on grindability and cement performance. Constr. Build. Mater..

[42] Liu X., Ye Z., Zhang L., Hou P., Cheng X. (2017). The influence of ethanol-diisopropanolamine on the hydration and mechanical properties of Portland cement. Constr. Build. Mater..

[43] Mhammed S., Safiullahb O. (2018). Optimization of the SO3 content of an Algerian Portland cement: Study on the effect of various amounts of gypsum on cement properties. Constr. Build. Mater..

